# Generation of anisotropic strain dysregulates wild-type cell division at the interface between host and oncogenic tissue

**DOI:** 10.1016/j.cub.2021.05.023

**Published:** 2021-08-09

**Authors:** Megan Moruzzi, Alexander Nestor-Bergmann, Georgina K. Goddard, Nawseen Tarannum, Keith Brennan, Sarah Woolner

**Affiliations:** 1 Wellcome Trust Centre for Cell-Matrix Research, Division of Cell Matrix Biology and Regenerative Medicine, School of Biological Sciences, Faculty of Biology, Medicine & Health, Manchester Academic Health Science Centre, University of Manchester, Oxford Road, Manchester M13 9PT, UK; 2 School of Mathematics, University of Manchester, Manchester M13 9PL, UK; 3 Division of Cancer Sciences, School of Medical Sciences, Faculty of Biology, Medicine and Health, University of Manchester, Manchester Academic Health Science Centre, Manchester M13 9PL, UK

**Keywords:** tissue mechanics, cancer, cell division, Xenopus, Ras, MYC, vertex model

## Abstract

Epithelial tissues are highly sensitive to anisotropies in mechanical force, with cells altering fundamental behaviors, such as cell adhesion, migration, and cell division.^1^, ^2^, ^3^, ^4^, ^5^ It is well known that, in the later stages of carcinoma (epithelial cancer), the presence of tumors alters the mechanical properties of a host tissue and that these changes contribute to disease progression.^6^, ^7^, ^8^, ^9^ However, in the earliest stages of carcinoma, when a clonal cluster of oncogene-expressing cells first establishes in the epithelium, the extent to which mechanical changes alter cell behavior in the tissue as a whole remains unclear. This is despite knowledge that many common oncogenes, such as oncogenic Ras, alter cell stiffness and contractility.^10^, ^11^, ^12^, ^13^ Here, we investigate how mechanical changes at the cellular level of an oncogenic cluster can translate into the generation of anisotropic strain across an epithelium, altering cell behavior in neighboring host tissue. We generated clusters of oncogene-expressing cells within otherwise normal *in vivo* epithelium, using *Xenopus laevis* embryos. We find that cells in kRas^V12^, but not cMYC, clusters have increased contractility, which introduces radial stress in the tissue and deforms surrounding host cells. The strain imposed by kRas^V12^ clusters leads to increased cell division and altered division orientation in neighboring host tissue, effects that can be rescued by reducing actomyosin contractility specifically in the kRas^V12^ cells. Our findings indicate that some oncogenes can alter the mechanical and proliferative properties of host tissue from the earliest stages of cancer development, changes that have the potential to contribute to tumorigenesis.

## Highlights

• The oncogenes, kRas^V12^ and cMYC, have differing impacts on epithelial mechanics
• kRas^V12^ cells are hyper-contractile, generating anisotropic strain across tissue
• Wild-type tissue responds to kRas^V12^-induced strain by altering cell division
• Relieving hyper-contractility restores isotropy and divisions and reduces tumor growth

## Graphical abstract

**Figure:**
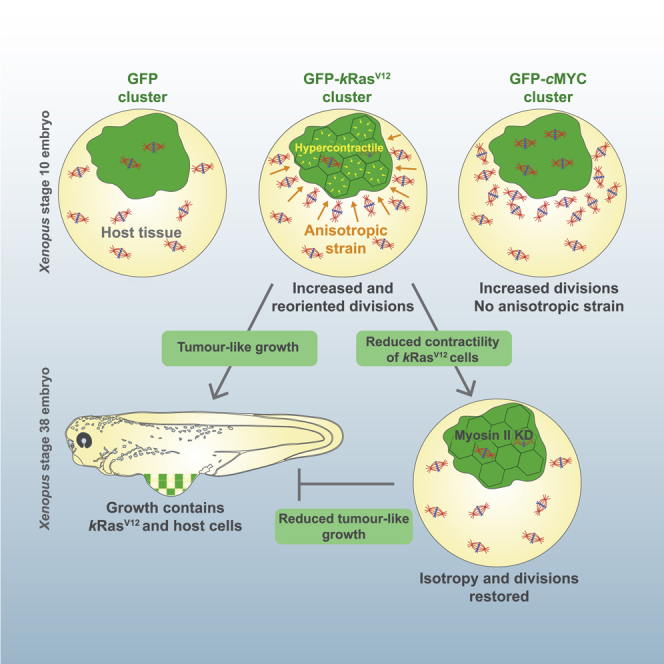

## Teaser

Moruzzi et al. show that clonal expression of a single oncogene alters tissue mechanics in host epithelium. kRas^V12^, but not cMYC, cells are hyper-contractile, generating anisotropic strain and driving increased and reoriented divisions in wild-type tissue. These effects are rescued, and tumor growth reduced, by relieving kRas^V12^ contractility.

## Results

### Modeling early-stage carcinoma in *Xenopus laevis*

**Figure 1:**
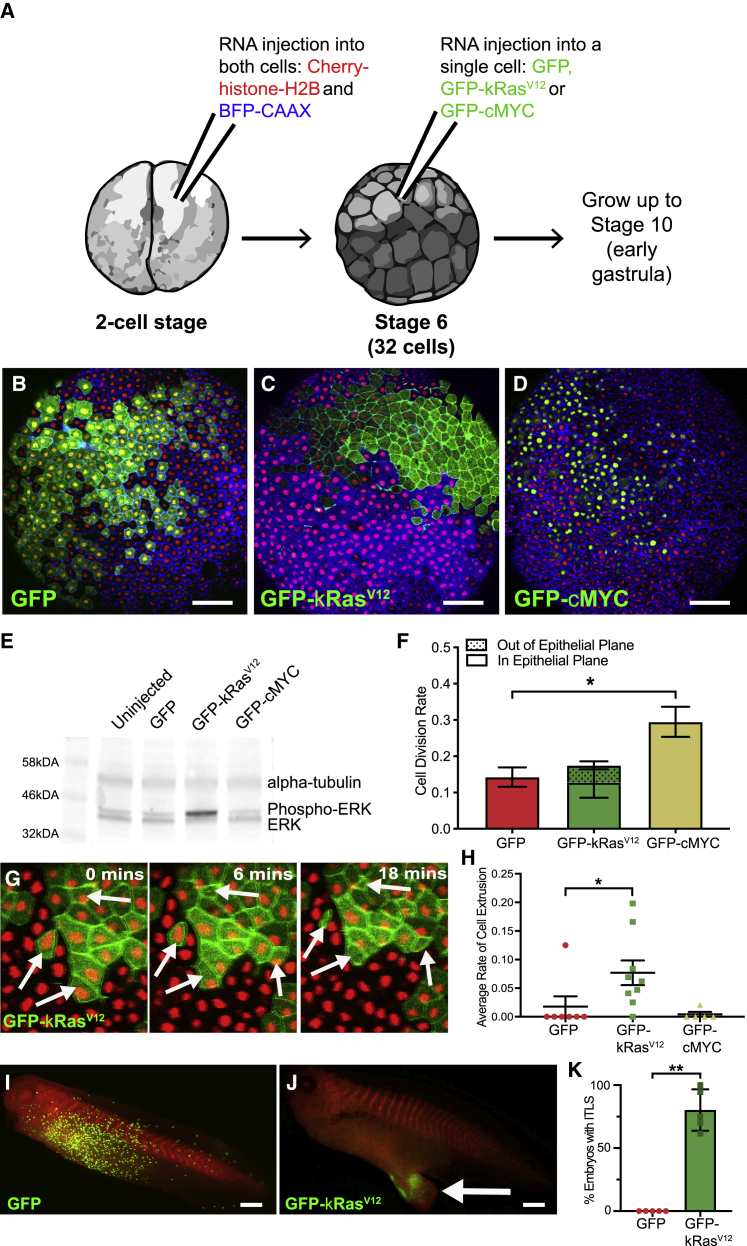
Modeling early-stage carcinoma in *Xenopus laevis* (A) Schematic of the microinjection protocol. *Xenopus* embryos were injected with Cherry-histone-H2B and BFP-CAAX mRNA at the 2-cell stage. At the 32-cell stage, a single cell was injected with GFP, GFP-kRas^V12^, or GFP-cMYC mRNA. Embryos were developed to early gastrula stage 10 and imaged. (B–D) Confocal microscopy images of *Xenopus* embryos developed to early gastrula stage 10, following injection of a single cell at the 32-cell stage with (B) GFP, (C) GFP-kRas^V12^, or (D) GFP-cMYC mRNA. Scale bars represent 100 μm. (E) Western blot showing phosphorylated ERK, unphosphorylated ERK, and α-tubulin expression in uninjected control embryos and embryos injected with GFP, GFP-kRas^V12^, or GFP-cMYC mRNA. (F) Bar chart showing the average percentage of cells that divided per minute of time lapse, in either GFP, GFP-kRas^V12^, or GFP-cMYC overexpression clusters (^∗^p < 0.05; Kruskal-Wallis test: n = 7 GFP, 8 GFP-kRas^V12^, and 9 GFP-cMYC embryos). Also displayed is the proportion of cell divisions that occurred out of the epithelial plane (shaded portion of the bar). Error bars show SEM. (G) Stills from a confocal microscopy time lapse of a representative embryo with a GFP-kRas^V12^ cell cluster at stage 10. White arrows highlight cells observed to be lost basally over the course of the time lapse. (H) Dot plot showing average percentage of cells that extruded basally from GFP, GFP-kRas^V12^, or GFP-cMYC cell clusters (^∗^p < 0.05; Kruskal-Wallis test: n = 7 GFP, 9 GFP-kRas^V12^, and 5 GFP-cMYC embryos). Error bars are SEM. (I and J) Microscopy images of representative embryos at stage 38 that had a (I) GFP- or (J) GFP-kRas^V12^-expressing cluster at stage 10. Arrow indicates an induced tumor-like structure (ITLS). Anterior is toward the left; scale bars represent 500 μm. (K) Quantification of ITLS formation at stage 38 in embryos that had GFP or GFP-kRas^V12^ clusters at stage 10 (^∗∗^p < 0.01; Mann-Whitney test; n = 5 independent experiments; a total of 120 GFP and 97 GFP-kRas^V12^ embryos were assessed). Error bars are SEM. See also Figure S1 and Videos S1 and S2.

To investigate how mechanical changes might alter cell behavior in a model of early-stage carcinoma, we chose two common oncogenes: kRas^V12^ and cMYC. Ras guanosine triphosphatases (GTPases) are known to sit upstream of actomyosin contractility. Constitutively active Ras mutations alter cell stiffness, although the effects vary between softening and stiffening cells,^11^^,^^12^^,^^14^ and increase junctional tension at the boundaries of Ras mutant clones in *Drosophila* epithelia.^10^ In contrast, MYC overexpression upregulates proliferation, increasing compressive forces in a tissue,^15^^,^^16^ and decreases junctional tension.^10^ To produce a cluster of oncogene-expressing cells within *in vivo* epithelial tissue, GFP-kRas^V12^ or GFP-cMYC mRNA was injected into a single cell of a 32-cell *Xenopus laevis* embryo (Figure 1A). By early gastrula stage, a cluster of GFP-expressing cells consistently developed in the superficial layer of the animal cap epithelium (Figures 1B–1D, S1A, and S1B).

The kRas^V12^ construct was confirmed functional, with expression increasing ERK phosphorylation (Figures 1E and S1C). As expected, cMYC significantly increased cell division rate (CDR) in the cluster (p < 0.5; Figure 1F). Surprisingly, kRas^V12^ did not increase CDR but increased the propensity for division out of the epithelial plane (Figures 1F and S1D).^17^^,^^18^ Contrasting to existing studies,^19^, ^20^, ^21^, ^22^ kRas^V12^ cells were not apically extruded (Figure S1E; Video S1) but were lost basally from the superficial layer (Figures 1G and 1H) at the edge of the cluster (72%: 18/25 cells, from 7 embryos). Imaging of fixed, bisected embryos revealed increased cell layers, increased animal cap thickness, and delamination of cells from the tissue (Figures S1F, S1G, and S4D).

Video S1. Time-lapse of kRas^V12^ cluster embryo, related to Figure 1 Stage 10 embryo with a GFP-kRas^V12^ (green) cluster shown in Figure S1E. Nuclei are visualized by cherry-H2B (red) and cell edges by BFP-CAAX (blue). Time stamp shows minutes.

Overexpression of cMYC can stimulate apoptosis,^23^, ^24^, ^25^, ^26^, ^27^ but live imaging and fixed staining with cleaved caspase-3 indicated no apoptosis in the superficial layer of embryos with cMYC clusters (Figures S1H–S1J; Video S2). However, significant evidence of apoptosis was observed in the deep, mesenchymal layer of cMYC animal caps, but not GFP or kRas^V12^ clusters (Figures S1I and S1J).

Video S2. Time-lapse of cMYC cluster embryo, related to Figure 1 Stage 10 embryo with a GFP-cMYC (green) cluster shown in Figure S1H. Nuclei are visualized by cherry-H2B (red) and cell edges by BFP-CAAX (blue). Time stamp shows minutes.

As described previously,^28^ induced tumor-like structures (ITLS) were formed by stage 38 in the majority (80% ± 7%) of embryos with kRas^V12^ clusters. Embryos with GFP or cMYC clusters remained morphologically normal (Figures 1J and 1K); however, almost no GFP-cMYC expression was observed at later stages (Figure S1K).^29^^,^^30^

### kRas^V12^ cell clusters have altered mechanical properties and impose strain on the tissue

**Figure 2:**
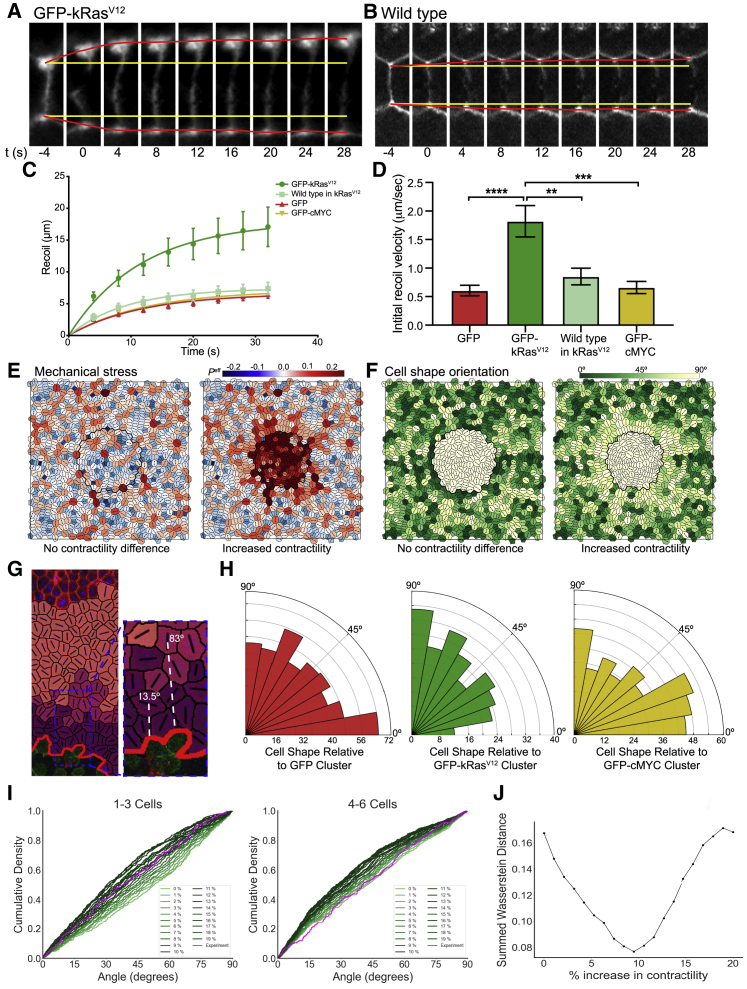
kRas^V12^ cell cluster imposes a mechanical strain on the wild-type epithelium (A and B) Cropped regions of confocal time-lapse stills showing laser ablation at a cell edge (highlighted by cherry-UtrCH: F-actin) in a GFP-kRas^V12^ cluster (A) and a surrounding wild-type cell (B). Ablation occurs at t = 0, yellow lines show the original positions of cell vertices before laser ablation, and red lines show the real-time positions of cell vertices. (C) Recoil measurements for cells in GFP-control (red), GFP-kRas^V12^ (green), and GFP-cMYC (yellow) clusters and areas of wild-type tissue around GFP-kRas^V12^ clusters (wild type; light green); n = 10 cells for each sample; error bars are SEM. (D) Initial recoil velocity calculated from recoil measurements in (C); one-way ANOVA: ^∗∗^p < 0.01; ^∗∗∗^p < 0.001; ^∗∗∗∗^p < 0.0001; n = 10 cells for each sample; error bars are SEM. (E) Simulated tissue, randomly generated, starting under conditions of zero net tissue stress. Heatmap indicates magnitude of cell-level isotropic stress, $P^{\text{eff}}$, with cells being under net tension (red) or compression (blue). A simulated Ras cluster was initialized in the center of the tissue (enclosed within black ring). Left: no additional contractility in cluster is shown. Right: 30% increase in cortical contractility, Γ, in cluster is shown. (F) Simulated tissues from (E), with heatmap showing the orientation of the principal axis of cell shape relative to the cluster (as shown in G). (G) From confocal images, the shapes of host cells neighboring the clusters (dark purple: 1–3 cells from cluster; light purple: 4–6 cells; pink: 7+ cells) were traced and cell shape orientation (long-axis) relative to the cluster was measured (two examples in white are shown). (H) Rose histograms showing the orientation of wild-type cells’ long axes 1–3 cells from GFP-control (red), GFP-kRas^V12^ (green), and GFP-cMYC (yellow) clusters, relative to the cluster, with the total number of cells analyzed across all embryos in each data group in 10° bins. Kruskal-Wallis test: GFP versus GFP-kRas^V12^ p < 0.01 and GFP versus GFP-cMYC p > 0.9999; n = 431 cells from 7 GFP embryos, 224 cells from 5 GFP-kRas^V12^ embryos, and 348 cells from 7 GFP-cMYC embryos. (I) Cumulative distributions of cell shape orientation relative to cluster (as shown in G), from experiments (magenta) and simulations (green). Ras clusters were simulated with varying degrees of increased cortical contractility, Γ. (J) Wasserstein distance between experiments and simulations for cumulative distributions in (I) and Figure S2E. Discrete intervals on the x axis relate to shades of green in (I). For every contractility interval, the y axis shows the sum of the Wasserstein distances over the three distance categories (1–3, 4–6, and 7+ cells). The best fit is found at a 9% increase in contractility, where summed Wasserstein distance is minimized. See also Figure S2.

We next investigated how the mechanical properties of the tissue might be altered. Cells expressing oncogenic Ras can be hyper-contractile, have altered stiffness, and exert increased traction forces on their substrate.^10^, ^11^, ^12^, ^13^^,^^19^ We tested how the mechanical properties of oncogene-expressing cells were altered by measuring recoil of junctional vertices following laser ablation of cell edges (Figures 2A–2D). The vertex-vertex initial recoil velocity (initial recoil) indicates junctional tension prior to ablation.^31^ We found that initial recoil within kRas^V12^ clusters was significantly higher than GFP or cMYC clusters or wild-type cells surrounding kRas^V12^ clusters (Figures 2A–2D), indicating higher contractility in kRas^V12^ clusters.

In *Drosophila*, there is increased tension specifically at the boundary between Ras and wild-type cells.^10^^,^^32^ To explore whether the increased tension in the kRas^V12^ cluster originated at the boundary, we performed junction ablations here. We found that both kRas^V12^ and wild-type junctions at the boundary showed an intermediate initial recoil, less than the recoil further within kRas^V12^ clusters but greater than the recoil further within wild-type tissue (Figures S2A and S2B), indicating that tension does not originate at the boundary. Because the fitting indicated that junction stiffnesses were equal (STAR Methods), these data suggest a direct increase in cortical contractility in kRas^V12^ cells that has a local effect on wild-type cells near the boundary, increasing their recoil in line with their kRas^V12^ neighbors.

We next explored how differences in contractility might affect the distribution of mechanical stress and strain across the tissue. We adopted a vertex-based model of an epithelium (STAR Methods) to simulate an increase in cortical contractility within a cell cluster. This produced a net radial tensile stress oriented toward the cluster at the boundary between wild-type cells (Figure 2E), distorting wild-type cell shapes and orienting their long axis toward the cluster (Figure 2F). Because the model predicts that the principal axis of cell shape (the long-axis; STAR Methods) aligns exactly with the major axis of cell-level stress, for a cell with homogeneous and isotropic material properties,^33^, ^34^, ^35^ we used the long axis as an indicator of mechanical stress in our experimental data.

Following the predictions of the model, we measured wild-type cell shape around the clusters (Figure 2G). As predicted, cells around kRas^V12^ clusters had altered orientation. Wild-type cells up to three cells away were significantly more likely to be oriented toward the cluster (p < 0.01; Figure 2H), indicating a localized effect (Figures S2C–S2F). Measurable changes in cell shape were also limited to orientation, with no significant difference in apical cell area or circularity observed between kRas^V12^ cells and their wild-type neighbors or GFP cells (Figures S2G and S2H). In line with our recoil results showing no change in cMYC cell contractility (Figures 2C and 2D), equivalent wild-type cells in cMYC embryos showed no geometric bias (Figure 2H).

To further interrogate changes in mechanics and cell shape, we combined our experimental data with the vertex model to predict the contractility change required in kRas^V12^ cells to elicit the wild-type cell shape changes seen around kRas^V12^ clusters. Increased contractility in kRas^V12^ cells was simulated by gradually increasing a cortical contractility parameter from 0% to 20% (STAR Methods). Grouping cells into three categories by their distance from the cluster, we compared cumulative distributions of cell shape orientation, relative to the cluster, between simulations and experiments (Figures 2I and S2I). To determine which simulation best matched the experimental data, we calculated the Wasserstein distance between distributions and found that a 9% increase in cluster cortical contractility corresponded to our experimental data (Figure 2J). These simulations demonstrate that increased contractility can lead to a radial stress that generates an anisotropic strain in surrounding tissue, altering surrounding cell shapes.

Together, these data suggest that kRas^V12^ cells are more contractile than wild type, leading to cells around kRas^V12^ clusters being pulled and distorted. These distortions indicate that the presence of kRas^V12^, but not GFP or cMYC, leads to a radial stress across the epithelium that is oriented toward the cluster.

### Wild-type epithelium responds to oncogene-expressing clusters with altered cell division

**Figure 3:**
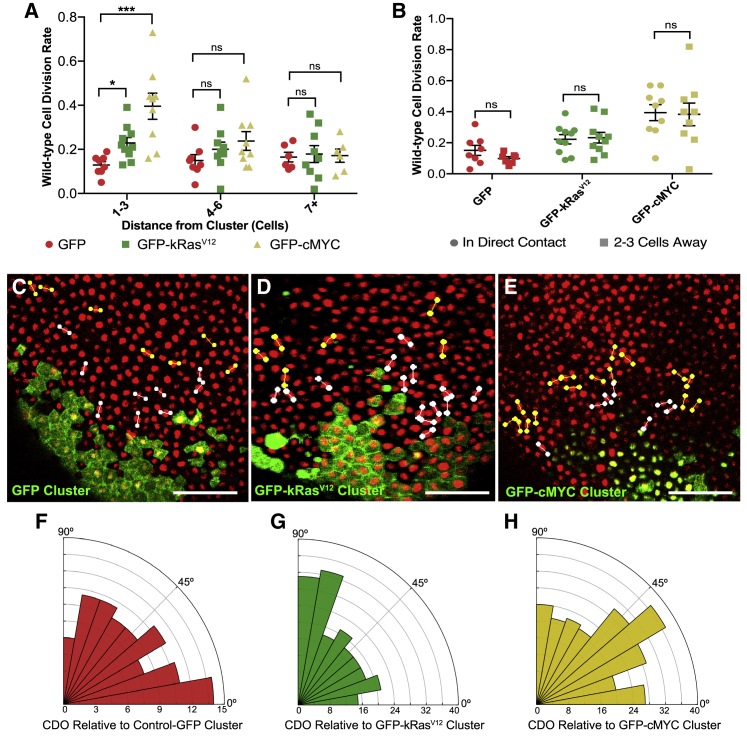
The wild-type epithelium responds to oncogene-expressing clusters with altered cell division (A and B) Dot plots showing the percentage of wild-type cells that divided per minute of time lapse at different distances from GFP, GFP-kRas^V12^, or GFP-cMYC clusters. (A) Kruskal-Wallis test: ^∗^p < 0.05 and ^∗∗∗^p < 0.001; n = 8 GFP-control, 10 GFP-kRas^V12^, and 9 GFP-cMYC embryos. Error bars are SEM. (B) Paired t tests were performed; n = 8 GFP, 10 GFP-kRas^V12^, and 9 GFP-cMYC embryos. (C–E) Snapshots from confocal microscopy time lapses of representative embryos showing the orientation of cell divisions that occurred in wild-type cells: colored lines were drawn, connecting the dividing anaphase nuclei, and are shown cumulatively for the entire time lapse in one snapshot. White lines label divisions 1–3 cells from the cluster, and yellow lines mark divisions 4–6 cells away. Scale bars represent 100 μm. (F–H) Rose histograms showing cell division orientation up to 6 cells away from (F) GFP control, (G) GFP-kRas^V12^, and (H) GFP-cMYC clusters, with the total number of cell divisions analyzed across all embryos in each data group in 10° bins. Kruskal-Wallis test: GFP versus GFP-kRas^V12^ p < 0.05 and GFP versus GFP-cMYC p > 0.9999; n = 88 divisions from 8 GFP embryos, 193 divisions from 11 GFP-kRas^V12^ embryos, and 231 divisions from 9 GFP-cMYC embryos. See also Figure S3.

Cell division is known to be sensitive to tension^1^^,^^36^, ^37^, ^38^, ^39^ and strain: stretching an epithelium increases CDR and reorients divisions along the axis of strain.^3^^,^^5^^,^^40^ Because the radial stress induced by kRas^V12^ clusters generates anisotropic strain, we hypothesized that this would affect cell division in the host tissue. Using time-lapse confocal microscopy, we found that wild-type cells up to three cells from kRas^V12^ showed significantly increased CDR (p < 0.05; Figure 3A). Surprisingly, considering we saw no significant effect on cortical contractility or cell shape (Figures 2C, 2D, and 2H), cMYC cell clusters stimulated an approximate 4-fold increase in surrounding wild-type CDR (p < 0.001; Figure 3A). In both kRas^V12^ and cMYC embryos, this CDR change was not a boundary-specific effect (Figure 3B), although it was localized to within six cells from the clusters (Figure 3A).

The orientation and frequency of cell division is usually tightly controlled within epithelial tissues.^41^ While the kRas^V12^ cells showed an increased propensity to divide out of the epithelial plane (Figure 1F), no wild-type cells up to three cells from kRas^V12^ or cMYC clusters were observed dividing out of the epithelial plane (data not shown; n = 10 GFP-*k*Ras^V12^ and 9 GFP-cMYC embryos). We quantified CDO within the epithelial plane by measuring the angle between the separating daughter nuclei at anaphase and the line from the cell centroid to the closest cluster edge (Figure S3A). Wild-type cells up to six cells from kRas^V12^ clusters had significantly altered CDO within the epithelial plane, compared with GFP embryos (p < 0.05; Figures 3F, 3G, and S3B–S3D). In contrast, wild-type CDO was not significantly altered in cMYC embryos (Figure 3H).

Given these changes in host cell division, we investigated whether wild-type cells contribute to the ITLS observed in later stage kRas^V12^ embryos. Cells that neighbored the GFP-kRas^V12^ mRNA-injected cells were injected with mCherry-H2B mRNA, and at early gastrula stage, embryos with a GFP-kRas^V12^ cell cluster surrounded by mCherry-H2B cells were selected (Figure S3E). At stage 38, kRas^V12^-driven ITLSs were analyzed and all growths were found to contain mCherry-H2B-expressing cells (39 ITLS, 6 independent experiments), demonstrating that cells derived from the host epithelium contributed to the tumor-like phenotype (Figures S3F and S3G).

These results show that the host epithelium displays altered cell division in response to groups of cells that overexpress kRas^V12^ or cMYC. In the case of kRas^V12^, the division effect is similar to that seen when an anisotropic strain is applied to an epithelial tissue: increased CDR and divisions oriented along the principal axis of shape.^3^ In contrast, cMYC clusters elicit only an increase in CDR, without perturbing cell shape or CDO.

### Activation of RhoA induces a response in wild-type epithelium comparable to kRas^V12^

**Figure 4:**
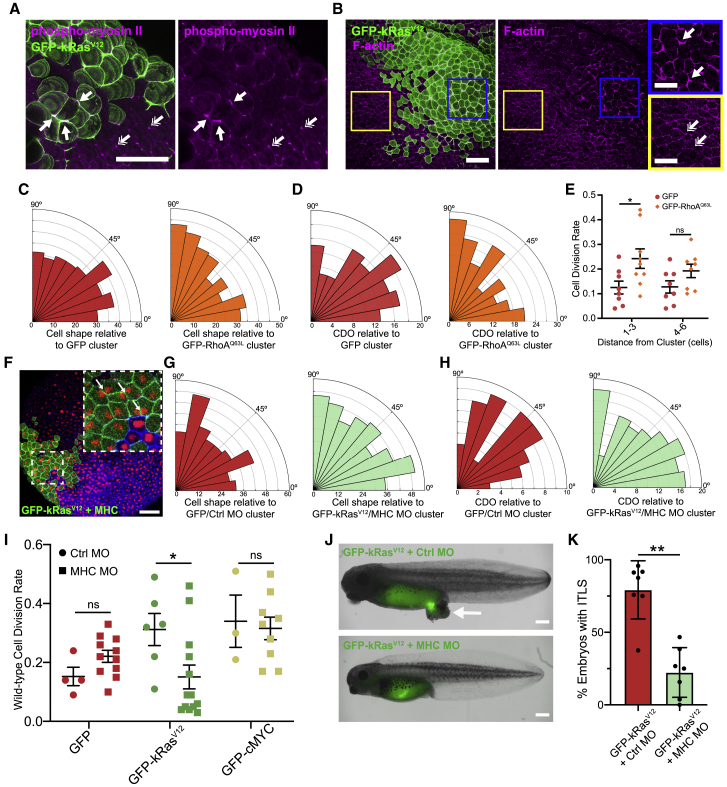
Actomyosin contraction in cluster is required to generate strain and alter cell division in wild-type tissue (A and B) Confocal images of fixed, stage 10 embryos with a GFP-kRas^V12^ cluster, stained for (A) phosphorylated myosin II (magenta), single-headed arrows highlight tricellular junctions with increased phospho-myosin II in GFP-kRas^V12^ cells compared to wild-type tissue (double-headed arrows), and (B) F-actin (phalloidin; magenta), single-headed arrows highlight increased F-actin at the cell cortex in the GFP-kRas^V12^ cluster compared to wild-type tissue (double-headed arrows). (C) Rose histograms showing the orientation of wild-type cells’ long axes up to 6 cells from GFP-control (red) or GFP-RhoA^Q63L^ (orange) cell clusters, relative to the cluster, in 10° bins. Kolmogorov-Smirnov test: p < 0.05; n = 298 cells from 6 GFP-control embryos and 299 cells from 6 GFP-RhoA^Q63L^ embryos. (D) Rose histograms showing cell division orientation relative to GFP-control (red) or GFP-RhoA^Q63L^ (orange) clusters, with the total number of cells in 10° bins. Kolmogorov-Smirnov test: p < 0.05; n = 98 divisions from 10 GFP-control embryos and 174 divisions from 9 GFP-RhoA^Q63L^ embryos. (E) Dot plot showing percentage of wild-type cells that divided per minute of time lapse at different distances from GFP-control or GFP-RhoA^Q63L^ clusters. One-way ANOVA: ^∗^p < 0.05; n = 7 GFP-control and 9 GFP-RhoA^Q63L^ embryos. Error bars are SEM. (F) Confocal microscopy image shows a myosin-II-deficient GFP-kRas^V12^ cell cluster. Arrows highlight “butterfly nuclei.” (G) Rose histograms showing the orientation of wild-type cell long axes up to 3 cells from GFP/Ctrl MO (red) or myosin-II-deficient (MHC MO) GFP-kRas^V12^ (light green) cell clusters, in 10° bins. Kruskal-Wallis test: p > 0.9999; n = 325 cells from 6 GFP/Ctrl MO embryos and 368 cells from 7 GFP-kRas^V12^/MHC MO embryos. (H) Rose histograms show cell division orientation up to 6 cells from (D) GFP/Ctrl MO (red) or GFP-kRas^V12^/MHC MO (light green) cell clusters, in 10° bins. Kruskal-Wallis Test: p = 0.9327; n = 58 divisions from 6 GFP/Ctrl MO embryos and 132 divisions from 9 GFP-kRas^V12^/MHC MO embryos. (I) Dot plot shows percentage of wild-type cells that divided per minute of time lapse, up to 3 cells from GFP, GFP-kRas^V12^, or GFP-cMYC control morpholino clusters or myosin-II-deficient GFP, GFP-kRas^V12^, or GFP-cMYC clusters. Kruskal-Wallis test: ^∗^p < 0.05; n = 5 GFP/Ctrl MO embryos, 11 GFP/MHC MO, 6 GFP-kRas^V12^/Ctrl MO, 13 GFP-kRas^V12^/MHC MO, 3 GFP-cMYC/Ctrl MO, and 9 GFP-cMYC/MHC MO embryos. (J) Images of representative embryos at stage 38 selected for presence of GFP-kRas^V12^ clusters at stage 10 and co-injected at 32-cell stage with Ctrl MO or MHC MO. Arrow indicates formation of ITLS in GFP-kRas^V12^/Ctrl MO embryo, but not GFP-kRas^V12^/MHC MO. (K) Quantification of ITLS formation at stage 38 in kRas^V12^/Ctrl MO and GFP-kRas^V12^/MHC MO embryos (p < 0.01; Mann Whitney test; n = 7 independent experiments; a total of 163 GFP-kRas^V12^/Ctrl MO and 124 GFP-kRas^V12^/MHC MO embryos were assessed). Error bars are SEM. Scale bars represent 100 μm in (A), (B) (main image), and (F); 50 μm in (B) (zoom-ins); and 500 μm in (J). See also Figure S4.

Anisotropic stress and strain can be generated when neighboring tissues with higher actomyosin contractility exert pulling forces.^1^^,^^42^, ^43^, ^44^, ^45^ Expression of oncogenic Ras stimulates cells to exert increased traction forces on their substrate in a Rho and non-muscle myosin-II-dependent manner.^13^ Fittingly, we found increased active, phosphorylated, myosin II in kRas^V12^ cells, which was especially prominent at tricellular vertices (Figures 4A and S4A). Furthermore, in kRas^V12^ clusters, F-actin organization was less homogeneous, with an increase in cortical actin close to tricellular vertices (Figure 4B). Importantly, we saw no evidence of an actomyosin cable around the cluster, making a wound-healing-like response unlikely.^10^^,^^43^^,^^46^, ^47^, ^48^ Similarly, no boundary effect was observed in terms of cell-cell adhesion, with no significant differences in C-cadherin (most abundant cadherin at stage 10) seen between kRas^V12^ cells and their wild-type neighbors (Figure S4B).

Myosin II is phosphorylated downstream of RhoA.^49^, ^50^, ^51^, ^52^, ^53^ To examine whether activation of RhoA can induce anisotropic strain in surrounding wild-type tissue, a group of cells were generated expressing the constitutively active RhoA^Q63L^ mutant.^54^^,^^55^ Similar to kRas^V12^ clusters, wild-type cells up to three cells from RhoA^Q63L^ clusters oriented their long axes toward the cluster (p < 0.05; Figure 4C) and wild-type CDO up to six cells from RhoA^Q63L^ clusters oriented toward the cluster (p < 0.05; Figure 4D). CDR was also significantly increased in wild-type cells up to three cells from RhoA^Q63L^ clusters (p < 0.05; Figure 4E). These results demonstrate increased RhoA activity is sufficient to induce cell shape changes in the surrounding wild-type epithelium and alter wild-type cell division in a similar manner to kRas^V12^ clusters. RhoA^Q63L^ clusters also caused a significant thickening of the animal cap tissue (Figure S4C) but did not develop ITLS (data not shown: 0 ITLS from 94 RhoA^Q63L^ embryos, 4 independent experiments).

### Non-muscle myosin II is required in kRas^V12^ clusters to alter wild-type tissue mechanics and cell division

Non-muscle myosin II is required for epithelial cells to generate contractile forces.^56^, ^57^, ^58^, ^59^, ^60^ To test whether kRas^V12^ cell contractility induced the observed anisotropic strain in the surrounding wild-type epithelium, we knocked down myosin II in only kRas^V12^ cells, through co-injection of a well-described morpholino (myosin heavy chain B [MHC] MO).^60^^,^^61^ The presence of butterfly-shaped nuclei in the kRas^V12^ clusters, signifying a mild cytokinesis phenotype, indicated reduced myosin II (Figure 4F). Myosin II knockdown did not significantly affect animal cap thickness around kRas^V12^ clusters (Figure S4D) or the CDR of kRas^V12^ cells (Figure S4E), although the length of mitosis was significantly longer (p < 0.0001; Figure S4F). Crucially, when myosin II was knocked down in the kRas^V12^ cells, wild-type cell orientation, up to three cells from kRas^V12^ clusters, was no longer significantly different to equivalent cells in control embryos (Figure 4G). Therefore, myosin II knockdown in the kRas^V12^ cells recovered cell shape isotropy in the surrounding wild-type epithelium, indicating restoration of isotropic stress. Myosin II knockdown in GFP or cMYC clusters had no effect on cell shape (Figures S4G and S4H).

When myosin II was knocked down in kRas^V12^ cells, CDO in surrounding wild-type cells was no longer significantly different to equivalent cells in control embryos (p < 0.05; Figure 4H). Myosin II knockdown had no effect on wild-type CDO in GFP or cMYC clusters (Figures S4I and S4J). The CDR of wild-type cells close to myosin-II-deficient kRas^V12^ clusters was significantly reduced (p < 0.05; Figure 4I). In contrast, myosin II knockdown in cMYC clusters did not significantly affect wild-type CDR (p = 0.7908; Figure 4I). To investigate the downstream consequence of myosin II knockdown in kRas^V12^ clusters, we developed embryos to stage 38 and assessed the formation of ITLS. We found a substantial and significant reduction in ITLS growth from myosin-II-deficient kRas^V12^ clusters (Figures 4J and 4K).

These data indicate that myosin II is required in kRas^V12^ cells in order for a kRas^V12^ cluster to generate anisotropic strain in the surrounding host epithelium, leading to increased CDR and altered CDO. Tissue isotropy and normal division behaviors are restored in the host tissue if myosin II is depleted in kRas^V12^ cells and ITLS formation is reduced.

## Discussion

In conclusion, we find that kRas^V12^ clusters in an otherwise normal epithelium generate localized anisotropic strain oriented toward the cluster. Anisotropic strain is known to alter cell division dynamics,^3^^,^^5^^,^^40^ and consistent with this, we see increased CDR and altered CDO in wild-type cells around kRas^V12^ clusters. We find that the anisotropic strain is caused by a radial tension, produced by greater actomyosin contractility in kRas^V12^ cells relative to cells in the surrounding epithelium. Isotropy and normal cell division can be recovered in wild-type host tissue when contractility in the kRas^V12^ cells is reduced by knockdown of myosin II. The correlation we see between rescue of normal cell division dynamics in host tissue and the reduction in ITLS formation upon myosin II knockdown is intriguing, especially given that no change was seen in the kRas^V12^ CDR or animal cap thickness when myosin II was depleted. Further analysis, including long-term tracking of host cell division and basal delamination in and around the cluster, will be required to determine how myosin II knockdown prevents ITLS formation.

We find that host CDR is significantly increased around cMYC clusters, while CDO and cell shape remained unaffected. Myosin II knockdown in the cMYC cells did not recover surrounding wild-type CDR, suggesting a distinct mechanism compared to kRas^V12^. Because previous studies have shown that cMYC overexpression inhibits the secretion of anti-mitotic factors,^62^^,^^63^ a possibility is that the host cells are responding to changes in their chemical, rather than mechanical, environment.

These results indicate novel roles for kRas and cMYC in inducing and dysregulating cell division in a host epithelium. An exciting avenue for future research is to determine whether the same responses occur in differentiated, adult tissues during carcinoma onset. The dysregulation of wild-type cell division in host epithelia could help drive the increase in cell number that defines early cancer stages and aid the spread of oncogenic cells through epithelial crowding and cell delamination.^2^^,^^64^ Moreover, faster and dysregulated divisions in the host tissue could increase the chance of these cells acquiring genetic changes of their own, increasing tumor heterogeneity and making this co-opting of the host epithelium a potential target for future therapeutic interventions.^65^

## STAR★Methods

### Key resources table

**Table:** 

REAGENT or RESOURCE	SOURCE	IDENTIFIER
**Antibodies**		

GFP Monoclonal (GF28R)	Thermo Fisher Scientific/Invitrogen	Cat# MA5-15256
GFP Polyclonal	Thermo Fisher Scientific/Invitrogen	Cat# A11122; RRID: AB_221569
Phospho-myosin light chain 2 (S19)	Cell Signaling	Cat# 3671; RRID: AB_330248
Cleaved Caspase-3 (Asp175)	Cell Signaling	Cat# 9661; RRID: AB_2341188
C-cadherin	Developmental Studies Hybridoma Bank	6B6
Phospho-ERK1/2	Sigma Aldrich	Cat# E7028; RRID: AB_259347
ERK1/2	Cell Signaling	Cat# 9102S; RRID: AB_330744
α-tubulin	Sigma Aldrich	Cat# T9026; RRID: AB_477593
Alexa Fluor 488 goat anti-mouse	Thermo Fisher Scientific/Invitrogen	Cat# A11001; RRID: AB_2534069
Alexa Fluor 568 goat anti-mouse	Thermo Fisher Scientific/Invitrogen	Cat# A11004; RRID: AB_2534072
Alexa Fluor 488 goat anti-rabbit	Thermo Fisher Scientific/Invitrogen	Cat# A11008; RRID: AB_143165
Alexa Fluor 568 goat anti-rabbit	Thermo Fisher Scientific/Invitrogen	Cat# A11011; RRID: AB_143157
Alexa Fluor 568 Tyramide Reagent	Thermo Fisher Scientific/Invitrogen	Cat# B40956
Goat anti-Rabbit IRDye800CW	Abcam	Cat# 216773
donkey anti-mouse IRDye680RD	Abcam	Cat# 216778

**Bacterial and virus strains**		

Subcloning Efficiency DH5α Competent Cells	Thermo Fisher Scientific	18265017

**Chemicals, peptides, and recombinant proteins**		

PMSG-Intervet (Pregnant Mare Serum Gonadotrophin)	Intervet UK	N/A
Chorulon (Human Chorionic Gonadotrophin)	Intervet UK	N/A
MS222 – Ethyl 3-aminobenzoate methanesulfonate salt	Merck	A5040-100G
Phenol:Chloroform:IAA, 25:24:1	Thermo Fisher Scientific	AM9730
L-cysteine	Sigma Aldrich	168149
Ficoll	Sigma Aldrich	PM400
Protease inhibitor cocktail	Promega	G6521
PhosSTOP phosphatase inhibitor	Sigma Aldrich	4906845001
DAPI	Thermo Fisher Scientific	D1306
NotI	New England Biolabs	R0189L

**Critical commercial assays**		

mMessage mMachine SP6 transcription kit	Thermo Fisher Scientific	AM1340
PureLink Quick Plasmid Miniprep Kit	Thermo Fisher Scientific	K210010

**Experimental models: Organisms/strains**		

Mature female *Xenopus laevis*	Bred in-house and from European *Xenopus* Resource Centre (EXRC).	https://xenopusresource.org/
Mature male *Xenopus laevis*	Bred in-house and from European *Xenopus* Resource Centre (EXRC).	https://xenopusresource.org/

**Oligonucleotides**		

Morpholino: MHC-B (Myosin Heavy Chain-B, myosin II) 5′-CTTCCTGCCCTGGTCTCTGTGACAT-3′	Gene Tools LLC^60^	N/A
Morpholino: Standard control 5′-CCTCTTACCTC AGTTACAATTTATA-3′	Gene Tools LLC	Product name “Standard Control oligo”
kRas^V12^FwdBspEI: 5′-CTGATCCGGAATGACTGAATATAAACTTGT-3′	Eurogentec	N/A
kRas^V12^RvsXhoI: 5′-GCTACTCGAGTTACATAATTACACACTTTG-3′	Eurogentec	N/A
MycFwdBspEI: 5′-AATATCCGGAATGCCCCTCA-3′	Eurogentec	N/A
MycRvsXhoI: 5′-ATACCTCGAGTTACGCACAAGAGTTC-3′	Eurogentec	N/A

**Recombinant DNA**		

mCherry-Histone2B in pCS2+	Kanda et al.^66^	N/A
BFP-CAAX in pCS2+	Bement Lab, University of Wisconsin-Madison.^67^	N/A
N-GFP in pCS2+	Bement Lab, University of Wisconsin-Madison^68^	N/A
GFP-kRas^V12^ in pCS2+	Human kRas^V12^ from Addgene cloned into N-GFP/pCS2+	Addgene 2544
GFP-cMYC in pCS2+	Human cMYC-IRES-GFP from Addgene cloned into N-GFP/PCS2+.	Addgene 8119
GFP-RhoA^Q63L^ in pcDNA3	Addgene	Addgene 12968
mCherry-UtrCH in pCS2+	Bement Lab, University of Wisconsin-Madison^68^	N/A

**Software and algorithms**		

Fiji/ImageJ Version 2.0.0-rc-69/1.53a	NIH	https://imagej.nih.gov/ij/
GraphPad Prism 9	GraphPad Software	https://www.graphpad.com/scientific-software/prism/
Python v3.6.5 – in-house python scripts implementing watershed algorithm.	Python Core Team	https://www.python.org/
Vertex-based model	Nestor-Bergmann et al.^3^^,^^69^	N/A

### Resource availability

#### Lead contact

Further information and requests for resources and reagents should be directed to and will be fulfilled by the lead contact, Sarah Woolner (sarah.woolner@manchester.ac.uk).

#### Materials availability

Plasmids generated in this study are available upon request from the lead contact.

#### Data and code availability

The published article includes all datasets generated or analyzed during this study. Data processing scripts and implementation of the vertex-based model are available upon request from the lead contact.

### Experimental model and subject details

#### Xenopus laevis

Female *Xenopus laevis* were housed within tanks maintained by the in-house animal facility at the University of Manchester. These females were used for embryo collection only. Female frogs were pre-primed 4-7 days in advance of egg collection with 50 U of Pregnant Mare’s Serum Gonadotrophin (Intervet UK) injected into the dorsal lymph sac. Four to seven days later, frogs were then primed with 500 U of Human Chorionic Gonadotrophin (Intervet UK) injected into the dorsal lymph sac.^70^ Primed frogs were maintained in individual tanks containing Marc’s modified Ringer’s (MMR; 100mM NaCl, 2mM KCl, 1mM MgCl2, and 5mM HEPES, pH 7.4). Eggs were collected from tanks 2-5 hours later and *in vitro* fertilization was performed. Male frogs were only used for testis extraction (in which males were euthanized by injection of MS222 (Tricaine) into the dorsal lymph sac to induce terminal anesthesia). All *Xenopus* work was performed using protocols approved by the UK Government Home Office and covered by Home Office Project License PFDA14F2D (License Holder: Professor Enrique Amaya) and Home Office Personal Licenses held by Sarah Woolner, Megan Moruzzi, Georgina Goddard and Nawseen Tarannum.

### Method details

#### Oncogene constructs

Human kRas^V12^ and cMYC were used in these experiments (Key resources table). kRas is 82% conserved at the mRNA level between *Xenopus* and mammals, with the proteins encoded sharing highly similar structures.^71^ cMYC is also highly conserved across vertebrates, including *Xenopus*^72^ and human cMYC has previously been demonstrated to rescue phenotypes induced in *Xenopus* when endogenous cMYC function is abrogated.^73^ Both constructs are also fusion proteins, N-terminally tagged with GFP (Key resources table). kRas had been N-terminally tagged in numerous studies, with no apparent consequences on its functionality.^74^ cMYC has also been N-terminally tagged with GFP in numerous studies, with one study showing GFP-cMYC can functionally replace endogenous cMYC in mice.^75^

#### mRNA Synthesis

Plasmids were linearized by restriction enzyme digestion. The resultant linearized DNA was the purified by a phenol/chloroform extraction and *in vitro* capped mRNA synthesis was carried out according to manufacturer’s instructions *(Ambion, #AM1340).* mRNA was then purified by a phenol/chloroform extraction. mRNA was diluted to 1 μg/μl and stored at −80°C until use.

#### *In vitro* Fertilization

*In vitro* fertilization was performed as described previously.^70^ MMR was removed from the collected eggs. A small amount of testis prep was cut up and spread over collected eggs to ensure all were exposed. After 5 mins at RT, the dish was topped up with 0.1X MMR and left for a further 30 mins. MMR was then drained and the embryos transferred into a glass beaker. 50 mL of 2% L-cysteine solution (2 g L-cysteine *(Sigma Aldrich, #168149-100G)* in 100 mL 0.1% MMR, pH 7.8 - 8.0) was added, and swirled gently until the jelly coat of the embryos was reduced. The L-cysteine solution was removed and the embryos washed a minimum of six times, with a total 200 mL 0.1% MMR. The embryos were transferred into new 10 mL Petri dish and topped up with fresh 0.1% MMR then incubated at RT to reach 2-cell stage.

#### mRNA Microinjection

Microinjections were carried out using Picospritzer lll Intracel injector (Parker instrumentation). Healthy embryos at the 2-cell stage were transferred into an injection dish containing 0.1X MMR with 5% Ficoll *(SigmaAldrich, #PM400)*. Each cell was injected with a total volume of 4.2 nL (for constructs and concentrations injected, see Table S1). Following this microinjection, embryos were washed in a Petri dish containing 0.1% MMR, then transferred into a second Petri dish containing fresh 0.1% MMR. These embryos were left at RT to develop to the 32-cell stage. At the 32-cell stage, the embryos were transferred back into the injection dish, containing 0.1% MMR and 0.5% Ficoll, and cells at the animal pole were injected with a total volume of 2.1 nL (for constructs and concentrations injected, see Table S1 below). Following microinjection, the embryos were washed in a Petri dish containing 0.1% MMR and then transferred into a second Petri dish containing fresh 0.1% MMR and incubated at 16°C overnight.

#### Myosin ll Knockdown

Myosin ll was knocked down through microinjection of a Morpholino targeting non-muscle myosin ll heavy chain 2B (MHC) (Key resources table).^60^ Prior to microinjection, the Morpholino was heated for 10 minutes at 65°C and combined with GFP-kRas^V12^ mRNA. The final needle concentration of the morpholino was 0.2 μM. A single cell at the animal pole of the 32-cell embryo was injected with a total volume of 2.1 nL of the GFP-Ras^V12^ mRNA (above) and MHC Morpholino (or Standard Control Morpholino at the same concentration).

#### Embryo Survival and Cluster Quantification

Following microinjection, embryos were incubated at 16°C for 16 h. At stage 10^76^ embryos were screened for survival and for the presence of an apical GFP cluster.

#### Western Blotting

Embryos were injected into both cells at the two-cell stage with GFP, GFP-kRas^V12^ or GFP-cMYC and grown to stage 10. Embryos were then washed three times in PBS and lysed by pipetting up and down in 10 μl ice-cold lysis buffer (Tris-HCl pH7.5, 150 mM NaCl, 0.5% NP-40, 5 mM EGTA, 5 mM EDTA) supplemented with 1X Protease inhibitor cocktail *(Promega G6521)* and PhosSTOP phosphatase inhibitor *(Sigma Aldrich)* per embryo. The embryos were then spun at 16873 x g for 15 mins at 4°C and the supernatant transferred into fresh tubes. Up to 10 μl of each sample was diluted with lysis buffer to make total volume of 15 μl. 5 μl of 4X loading buffer (8% SDS, 0.2 M tris-Cl pH 6.8, 8% Glycerol and 0.8% 2-mercaptoethanol) was added and the samples were incubated at 95°C for 10 mins. Samples were loaded into 4%–15% Mini-PROTEAN TGX Stain-Free Protein Gels *(Bio-Ra, #4568093)* and were fractionated by SDS-PAGE, before transfer to a 0.45 μm nitrocellulose membrane *(GE Healthcare, #10600002)* using a transfer apparatus according to the manufacturer’s protocols *(Bio-Rad)*. The membrane was blocked by incubation with 5% non-fat milk (or 5% BSA for phospho-specific antibodies) in TBST (10 mM Tris, pH 8.0, 150 mM NaCl, 0.5% Tween 20) for 1 h. Following this, the membrane was washed once with TBST and incubated with primary antibodies at 4°C for 12 h (Phospho-ERK1/2 1:500 *(Sigma Aldrich, #E7028)*; ERK1/2 1:1000 *(Cell Signaling, #9102S),* α-tubulin *(Sigma Aldrich, #T9026)*). The antibodies were diluted in the same solution that was used for blocking. Membranes were washed three times for 10 mins with TBST and incubated with IRDye conjugated antibodies (Goat anti-Rabbit IRDye800CW 1:5000 *(abcam, #216773)*, donkey anti-mouse IRDye680RD 1:5000 *(abcam, #216778)*), diluted in blocking solution. Membranes were then washed three times more and an Odyssey CLX LICOR was used to image the blot. To quantify western blots, band intensity was measured using Image Studio (LI-COR Biosciences). The intensity of each band was normalized to background fluorescence. Bands for total ERK and phopho-ERK were then normalized to the loading control (α-tubulin). The fold change relative to GFP control was then calculated.

#### Immunofluorescence

Embryos at stage 10 were fixed overnight with a gentle rotation at RT in fix: 3.7% fresh formaldehyde, 0.25% glutaraldehyde, 0.2% Triton X-100, 69.6 mM K-Pipes, 4.35 mM EGTA, 0.87 mM MgCl_2_. The following day, embryos were washed five times with PBS and the vitelline membranes were removed using forceps. Embryos were then quenched in 100 mM sodium borohydride in PBS for 2 h, rotating at RT. Embryos were washed three times in PBS for 5 mins and bleached for 90 mins in 10% H_2_O_2_ on a lightbox at RT. Embryos were washed three times for 10 minutes on a rotator in TBSN (Tris- buffered saline: 155 mM NaCl, 10 mM Tris-Cl [pH 7.4]; 0.1% Nonidet P-40) and then blocked overnight in 10 mg/ml BSA at 4°C with rotation. The block solution was changed twice the following day, and then primary antibodies were added at a dilution of 1:200: GFP monoclonal *(Invitrogen, MA5-15256)*, GFP polyclonal *(Invitrogen #A11122)*, phospho-myosin light chain 2 (S19) *(Cell Signaling, #3671*), cleaved Caspase-3 (Asp175) *(Cell Signaling #9661)* or 1:100: C-cadherin *(DSHB #6B6)* and incubated overnight at 4°C. The following day, embryos were washed five times in TBSN/BSA for 1 h at 4°C while rotating and incubated with secondary antibodies overnight at 4°C at a dilution of 1:400 (see Key resources table). Embryos were then washed three times in TBSN/BSA for 1 h at 4°C while rotating and then twice in TBSN alone for one hour at 4°C. Phospho-myosin light chain 2 was visualized using a Tyramide SuperBoost Kits according to manufacturers instructions. Nuclei were visualized by staining with DAPI at a dilution of 10 μg/ml (*Thermo-Scientific, #D1306*) and then washed three times in TBSN for half an hour at 4°C. After staining, samples were dehydrated in methanol, cleared and mounted in Murray’s Clear (2:1 benzyl benzoate:benzyl alcohol) and imaged using a Leica SP8 Confocal Microscope.

Phalloidin staining was carried out using albino embryos. Injected embryos were rinsed three times in PBS, and then fixed for 4 h at RT (3.7% formaldehyde, 0.25% glutaraldehyde and 0.1% Triton-X in PBS) while rotating gently. Embryos were washed three times in PBS and bisected along the sagittal axis using a razor blade and the vitelline membranes removed using forceps. The embryos were washed a further three times in PBTw (PBS + 0.1% Tween) and incubated overnight while rotating at 4°C in 0.005 U/μl Alexa Fluor 594 phalloidin (*Invitrogen, #A12381)* in PBTw. The following day, embryos were washed five times in PBS for 1 h while rotating at 4°C. After staining, samples were dehydrated in isopropanol, cleared and mounted in Murray’s Clear (2:1 benzyl benzoate:benzyl alcohol) and imaged using a Leica TPS SP8 AOBS inverted confocal microscope.

#### ITLS growth

To determine whether oncogene expression led to the formation of ITLS, clusters were generated as described and positive embryos with clear clusters selected at stage 10. Embryos were then developed to stage 38 and the presence of ITLS containing GFP-positive cells was visually assessed using a Zeiss Stereo Lumar microscope. To determine whether wild-type cells close to the initial oncogene-expressing cell contributed to the ITLS, cells that neighbored the GFP-kRas^V12^ injected cell at the 32-cell stage were injected with mCherry-H2B mRNA. At stage 10, embryos were screened and only those with mcherry-H2B cells surrounding but not within the GFP-kRas^V12^ cluster were selected and grown to stage 38. Images of the resulting ITLS were taken using a Leica M205 FA upright Stereomicroscope and the extent of wild-type contribution was categorized (high, medium, low).

#### Live Imaging

##### Stage 10

Approximately 21 hours after fertilization, when the embryos were at stage 10,^76^ they were transferred into fresh dish of 0.1 X MMR, which had 1 mm Polypropylene mesh (*SpectrumLabs, P/N146410*) stuck to its base to prevent the embryos rolling. Live-imaging was then performed using a dipping lens so as not to apply any mechanical stress by using a coverslip. Images were collected on a Leica TCS SP5 AOBS upright confocal using a 20x/0.50 HCX Apo U-V-I (Dipping Lens) objective and 1x confocal zoom. The confocal settings were as follows: pinhole 1 airy unit, scan speed 1000Hz bi-directional, format 512 × 512. Images were collected using the following detection mirror settings: BFP 406-483, eGFP 498-584 nm and mCherry 604-774 nm using the 405 nm, 488nm (25%) and 594nm (25%) laser lines respectively. Images were collected sequentially to eliminate bleed-through between channels. The distance between each optical stack was maintained at 4.99 μm and the time interval between each capture was 1 min, with each sample imaged for up to 1 h. The maximum intensity projections of these three-dimensional stacks are shown in the results.

##### Stage 38

Stage 38 embryos were transferred to a Petri dish containing a 0.4% MS222 anesthetic solution and imaged using a Leica M205 FA upright Stereomicroscope using a 1x / 0.10 PlanAPO objective and captured using a DFC 365FX (Leica) camera through LAS AF v3.1.0.8587 software (Leica). Specific band pass filter sets for GFP and mCherry were used to prevent bleed-through.

#### Laser ablation and recoil measurements

Images of *Xenopus laevis* embryos were acquired using a Nikon A1R confocal microscope using a 60x NA1.4-CFI-Plan-Apo oil objective and NIS-Elements software (Nikon). Laser ablation was performed using a Micropoint ablation laser (Andor Systems) attached to the Nikon confocal. The confocal settings were as follows: pinhole 1 airy unit, scan speed 400Hz unidirectional, format 512 × 512, 1x confocal zoom. GFP and mCherry-UtrCH were imaged using the 488nm and 561nm laser lines respectively. A single focal plane was captured for each embryo with a frame every 4 s for 2-3 minutes. A wounding laser level of 85 with a single blast setting was used to create a small wound at the cell edge. In order to provide a pre-ablation image and to visualize the moment of wounding, ablation was performed a few frames into the capture. A cell edge was targeted in one of four locations: 1. within the cluster (at least 3 cells from the boundary) 2. within the non-cluster, wild-type, region (at least 3 cells away from the cluster), 3. in cluster cells immediately adjacent to the cluster boundary (junctions perpendicular to border) 4. in wild-type cells immediately adjacent to the cluster boundary (junctions perpendicular to border).

### Quantification and statistical analysis

#### Quantification of immunofluorescence images

To quantify the number of apoptotic cells using cleaved caspase-3 staining, confocal z stacks were collected of GFP, GFP-kRas^V12^ and GFP-cMYC clusters and surrounding tissue. Using Fiji/ImageJ, each z stack was split into 3 grouped z-projections that contained only superficial cells (one z-projection) or only deep cells (two z-projections to cover multiple deep cell layers). In each z-projection the number of cleaved caspase-3 positive cells and total cells (DAPI positive) were counted using the “Cell Counter” plugin. The percentage of cleaved caspase-3 positive cells was then calculated for superficial and deep layers for each embryo.

To quantify the intensity of phospho-myosin II staining, confocal images were collected that included GFP-kRas^V12^ clusters and wild-type neighbors in a single image. Using Fiji/ImageJ, single wild-type or kRas^V12^ cells were selected using the “Freehand selections” tool to draw around single cells. Five cells were selected for wild-type and kRas^V12^ in each embryo and the mean gray value for each cell in the phospho-myosin II channel was measured and recorded. The mean intensity measurement for each sample of five cells was calculated and used to calculate a fold-change difference in intensity between wild-type and kRas^V12^ cells in each embryo.

For quantification of animal cap thickness, side-view images of bisected embryos were collected by confocal. In Fiji/ImageJ, the “Straight line” tool was used to draw a line measuring animal cap thickness from apical to basal. This was performed at three evenly spaced positions across the cluster and the mean calculated to give an animal cap thickness measurement for each embryo.

#### Initial Recoil Velocity

To determine initial recoil from the laser ablation movies we followed a previously described protocol.^31^ In brief, to measure the deformation of the cell junction following ablation, the xy coordinates of the two vertices (identified by mCherry-UtrCH) each side of the wound were tracked in ImageJ using the MTrackJ plugin. This data was used to extract the initial recoil and *k* (a ratio between junctional elasticity and viscosity of the cytoplasm) values, fitted to a Kelvin-Voigt model.^77^ No significant difference in *k* values was seen between any of the samples tested (data not shown), meaning that changes in initial recoil could be interpreted as an indication that junctional tension was affected.^31^ All initial recoil measurements were found to be normally distributed by Shapiro-Wilk test and were compared using a one-way ANOVA with Tukey’s multiple comparisons test.

#### Cell Division Analysis

Embryo time-lapse videos were generated using ImageJ64, from which snapshots were selected. Cell division rate in the epithelial plane was quantified as the percentage of cells where daughter nuclei were observed to separate, per minute. Cells that exhibited nuclear envelope breakdown, but where daughter nuclei were not observed to separate within the plane of the epithelium, were assumed to have divided out of plane. In plane CDO was measured using the ImageJ straight-line tool to draw a line between the dividing nuclei of a cell in anaphase and the closest edge of the cluster (see Figure S3A). Mitotic length was defined as the time between nuclear envelope breakdown and the first frame where daughter nuclei were observed to separate.

#### Cell Shape Analysis

Analysis of cell shapes was carried out by segmenting cells of interest, using an initial manual trace of cell edges. The principal axis of cell shape (described below) was calculated using a previously published in-house Python script.^3^^,^^69^ Cell shape was characterized by a shape tensor derived from the second moments of the positions of the tricellular junctions (we also include the rare case where more than three edges meet). For every cell we label the cell vertices i=1,2,…,n anticlockwise, where n is the number of vertices. The cell centroid, C, is the arithmetic mean of the positions of the vertices

$$C=\frac{1}{n}\sum_{i=1}^{n}R_{i}$$

where $R_{i}$ is the position vector of vertex i. The cell shape tensor, S, is then defined as

$$S=\frac{1}{n}\sum_{i=1}^{n}\left(R_{i}-C_{i}\right)⊗\left(R_{i}-C_{i}\right)$$

where ⊗ is the outer product. The principal axis of cell shape is defined as the eigenvector associated with the principal eigenvalue of S. The cell circularity, C, is defined as the ratio of the smaller eigenvalue over the larger eigenvalue. The circularity therefore takes values in the range (0, 1] (where a perfect circle gives a value of 1).

#### Simulations using a vertex-based model

Simulations were done in the framework of a vertex-based model, where the tissue is represented as a planar network of polygons. The model and simulation procedure are identical to our previously published methods.^3^^,^^69^^,^^78^ Briefly, we assume that every cell has a dimensionless mechanical energy, U, defined by

$$U=\;{\left(A-\;1\right)}^{2}+\frac{\text{Γ}}{2}{\left(L+\frac{\text{Λ}}{2\text{Γ}}\right)}^{2}$$

where while A and L denote the dimensionless area and perimeter of a cell, Γ is a cortical contractility and stiffness parameter and Λ is a mechanical parameter prescribing the preferred perimeter $L_{0}=-\text{Λ}/\left(2\text{Γ}\right)$. Mechanical equilibrium is found by minimizing the total mechanical energy, summed over all cells. For all simulations we use the parameters (Λ,Γ)=(0.259,0.172), which have previously been fitted to the *Xenopus* animal cap tissue.^69^ We simulate the effect of extra contractility in Ras clusters by inducing a percentage increase to the reference cortical stiffness parameter, Γ. Such a procedure has previously been shown to well-replicate the behavior of hyper-contractile tissues.^79^

As described in Nestor-Bergmann et al.,^69^ the magnitude of cell stress can be characterized by the isotropic component, $P^{\text{eff}}$, of the cell-level stress tensor:

$$P^{\text{eff}}=A-1+\frac{\text{Γ}L^{2}}{2A}-\frac{\text{Λ}L}{4A}$$

where positive values of $P^{\text{eff}}$ indicate that the cell is under net tension and negative values indicate net compression.

#### Statistical analysis

Rose histograms were generated using a python script and all other charts were produced using Prism 7/8 (GraphPad Software, LLC). Statistical analysis was performed using Prism 8. For cell shape and division orientation angle data, normality could not be assumed and individual comparisons were made using Kolmonov-Smirnov tests. For multiple comparisons of angle data, Kruskal-Wallis with Dunn’s tests were performed. For comparisons between paired division rate data at the boundary of the oncogenic cluster, distributions were found to be normally distributed by a Shapiro-Wilk test, and paired t tests were performed. For all other analyses, distributions were first tested for normality using Shapiro-Wilk tests. If normality was passed, single comparisons were made using unpaired Student t tests, while multiple comparisons were performed using one-way ANOVA with Sidak’s (for comparisons to a selected control) or Tukey’s (to compare every mean); if normality could not be assumed, single comparisons were made using Mann-Whitney tests, while multiple comparisons were performed using Kruskal-Wallis with Dunn’s post hoc test. For all statistical tests performed, n numbers and p values are given in the relevant figure legends.

## Supplemental information

Document S1. Figures S1–S4 and Table S1

Document S2. Article plus supplemental information
