# Supplementary material for: Generation of anisotropic strain dysregulates wild-type cell division at the interface between host and oncogenic tissue
Source: Curr Biol. 2021 Aug 9;31(15):3409–3418.e6. doi: 10.1016/j.cub.2021.05.023 (PMC8360906; doi:10.1016/j.cub.2021.05.023)
Supplement: Document S1. Figures S1–S4 and Table S1 [file mmc1.pdf]

Current Biology, Volume *31*

Supplemental Information

Generation of anisotropic strain dysregulates wild-type cell division at the interface between host and oncogenic tissue

Megan Moruzzi, Alexander Nestor-Bergmann, Georgina K. Goddard, Nawseen Tarannum, Keith Brennan, and Sarah Woolner

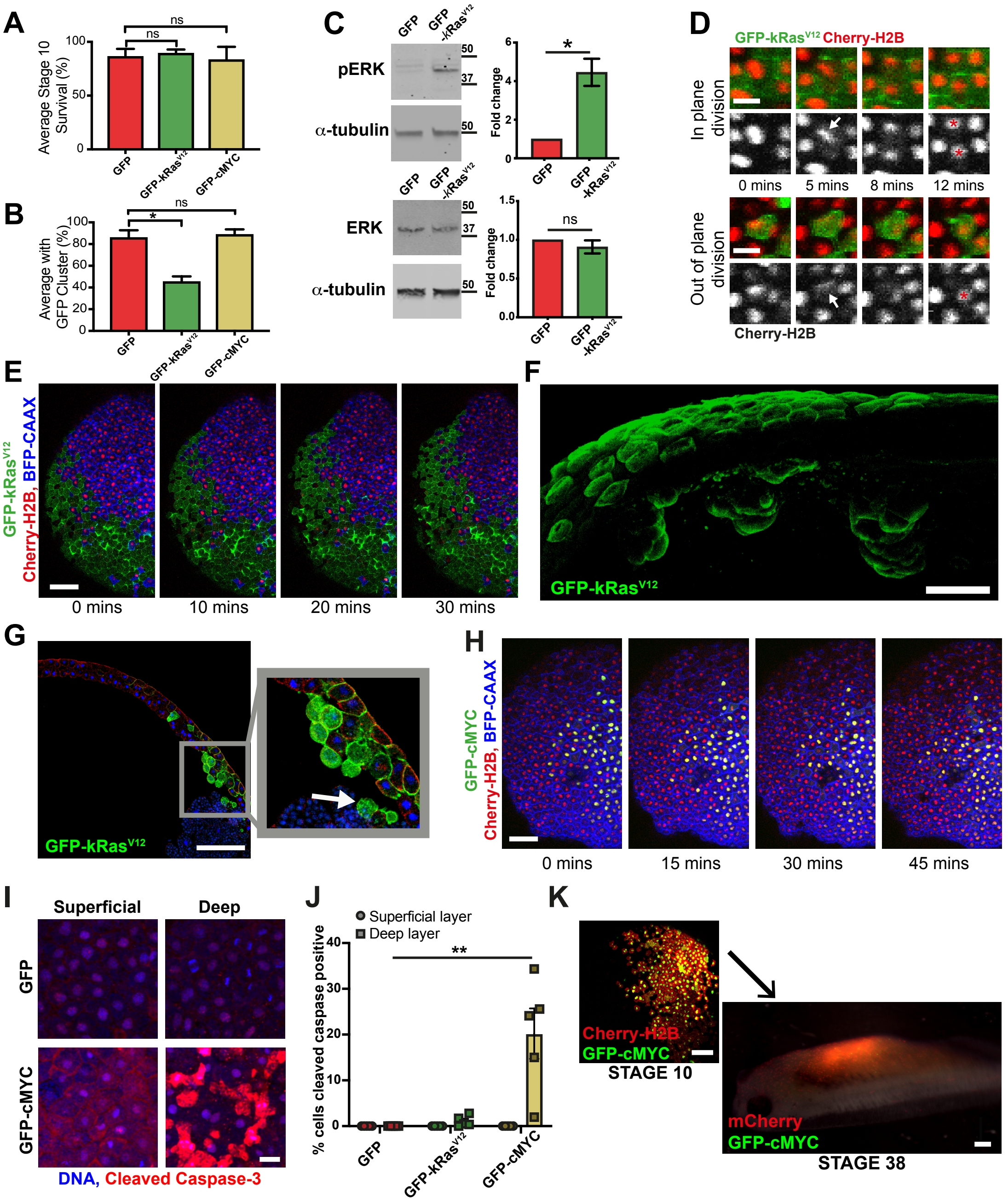

A
ns
ns
Average Stage 10 Survival (%)
100
50
0
GFP
GFP-kRas^V12
GFP-cMYC
B
ns
*
Average with GFP Cluster (%)
100
80
60
40
20
0
GFP
GFP-kRas^V12
GFP-cMYC
C
GFP
GFP -kRas^V12
pERK
α-tubulin
50
37
50
Fold change
6
4
2
0
*
GFP
GFP -kRas^V12
ERK
α-tubulin
50
37
50
Fold change
1.5
1.0
0.5
0.0
ns
D
GFP-kRas^V12 Cherry-H2B
In plane division
0 mins
5 mins
8 mins
12 mins
Out of plane division
Cherry-H2B
E
GFP-kRas^V12, Cherry-H2B, BFP-CAAX
0 mins
10 mins
20 mins
30 mins
F
GFP-kRas^V12
G
GFP-kRas^V12
H
GFP-cMYC, Cherry-H2B, BFP-CAAX
0 mins
15 mins
30 mins
45 mins
I
Superficial
Deep
GFP
GFP-cMYC
DNA, Cleaved Caspase-3
J
Superficial layer
Deep layer
**
% cells cleaved caspase positive
40
30
20
10
0
GFP
GFP-kRas^V12
GFP-cMYC
K
Cherry-H2B
GFP-cMYC
STAGE 10
mCherry
GFP-cMYC
STAGE 38

Figure S1: Further Characterisation of Oncogene-Expressing Cell Clusters in *Xenopus laevis*. Related to Figure 1.

(**A**) Bar chart shows the average percentage of embryos, injected at the 32-cell stage, alive at stage 10. Error bars show SEM. (**B**) Bar chart shows the average percentage of surviving stage 10 embryos, injected at the 32-cell stage, that have a GFP-positive cluster in the superficial animal cap layer (*$p<0.05$, Kruskal-Wallis test, n=3 clutches of embryos). Error bars show SEM. (**C**) Western blots (left) and associated quantification (right), showing phosphorylated ERK (pERK), ERK and α-tubulin expression in embryos injected with GFP or GFP-kRas^{V12}. For quantification, pERK and ERK levels were normalised against α-tubulin and shown as a fold change (*$p<0.05$, Student t-test, n=3 independent experiments). Error bars show SEM. (**D**) Classification of in-plane and out of plane divisions for data in Figure 1F. Divisions in GFP-kRas^{V12} (green) cells were identified in time-lapse videos by condensed chromosomes at the metaphase plate (arrows) using Cherry-H2B (red and single greyscale channel) and followed through to cytokinesis. If two nuclei could be seen separating and resolving in two cells in the epithelium the division was classified as "in-plane"; if only one nucleus resolved the division was classified as "out of plane" (nuclei following cytokinesis are marked with a red asterisk). (**E**) Stills from a representative confocal time-lapse of a *Xenopus* embryo at early gastrula stage 10, with a GFP-kRas^{V12} cell cluster in the superficial animal cap layer (Video S1). No apical extrusion or apoptosis was observed in either the GFP-kRas^{V12} clusters or the surrounding wild-type cells. (**F**) Confocal image shows an embryo, where GFP-kRas^{V12} mRNA was injected into a single cell at the 32-cell stage, that was fixed at stage 10, bisected and immunostained for GFP (green). (**G**) Confocal image shows an embryo, where GFP-kRas^{V12} mRNA was injected into a single cell at the 32-cell stage, that was fixed at stage 10, cryosectioned and immunostained for GFP (green), tubulin (red) and DAPI (blue). Arrows highlight cells that have lost cell-cell junctions and are no longer attached to the animal cap. (**H**) Stills from a representative confocal microscopy time-lapse of a *Xenopus* embryo at early gastrula stage 10, with a GFP-cMYC cell cluster in the superficial animal cap layer (Video S2). No apical extrusion or apoptosis was observed in either the GFP-cMYC clusters or the surrounding wild-type cells. (**I**) Immunofluorescence of cleaved caspase-3 (red; nuclei in blue) in GFP and GFP-cMYC injected embryos. Images of superficial and deep layers of the same stage 10 embryo are shown, cells positive for cleaved caspase-3, a marker of apoptosis, are found in the deep layer of GFP-cMYC injected embryos. (**J**) Quantification of cleaved caspase positive cells in GFP, GFP-kRas^{V12} or GFP-cMYC injected embryos ($p<0.01$, Kruskal-Wallis test, n=5 embryos). (**K**) Microscopy images show a representative embryo at stage 10 and stage 38 that was co-injected with GFP-cMYC and mCherry mRNA at the 32-cell stage. Anterior is towards the right. Scale bars represent 20 μm in D and I, 50 μm in F ,100μm in E, G, H and K (Stage 10), and 500μm in K (Stage 38).

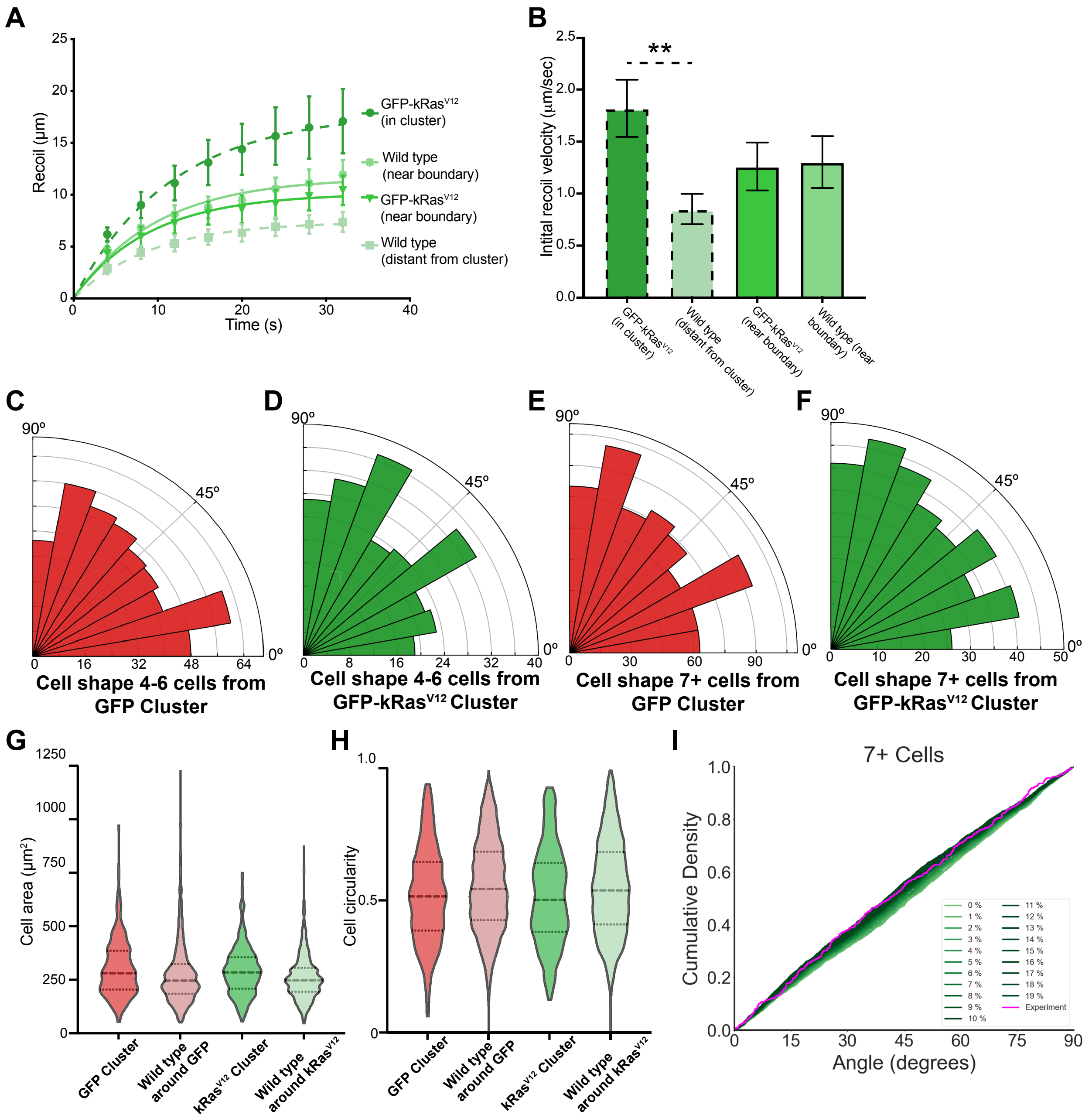

A
Recoil (µm)
Time (s)
GFP-kRas^{V12} (in cluster)
Wild type (near boundary)
GFP-kRas^{V12} (near boundary)
Wild type (distant from cluster)
B
Intital recoil velocity (µm/sec)
**
GFP-kRas^{V12} (in cluster)
Wild type (distant from cluster)
GFP-kRas^{V12} (near boundary)
Wild type (near boundary)
C
Cell shape 4-6 cells from GFP Cluster
D
Cell shape 4-6 cells from GFP-kRas^{V12} Cluster
E
Cell shape 7+ cells from GFP Cluster
F
Cell shape 7+ cells from GFP-kRas^{V12} Cluster
G
Cell area (µm²)
GFP Cluster
Wild type around GFP
kRas^{V12} Cluster
Wild type around kRas^{V12}
H
Cell circularity
GFP Cluster
Wild type around GFP
kRas^{V12} Cluster
Wild type around kRas^{V12}
I
7+ Cells
Cumulative Density
Angle (degrees)
Experiment

Figure S2: Analysis of mechanical strain and cell shape in and around kRas^{V12} clusters. Related to Figure 2.

(**A**) Recoil measurements for wild-type or GFP-kRas^{V12} cells adjacent to the cluster boundary (solid lines) compared to recoil within cluster and wild-type cells away from the boundary (dashed lines: data sets shown in Figure 2C); n=10 cells for each sample, error bars are SEM. (**B**) Initial recoil velocity calculated from recoil measurements in (A) with data from Figure 2D shown as comparison (dashed); One-way ANOVA: **p<0.01, no other difference was significant, n=10 cells for each sample, error bars are SEM. (**C-F**) Rose histograms show the orientation of wild-type cells long-axes 4-6 cells (C and D) and 7 or more (E and F) cells from GFP-control (C and E) or GFP-kRas^{V12} (D and F) cell clusters, relative to the cluster, with the total number of cell divisions that were analysed across all embryos each data group in 10° bins. Kruskal-Wallis test: 4-6 cells: p=0.1572, n=433 cells from 5 GFP-control embryos and 240 cells from 5 GFP-kRas^{V12} embryos. 7+ cells: p>0.9999, n=690 cells from 5 GFP-control embryos and 344 cells from 4 GFP-kRas^{V12} embryos. (**G-H**) Violin plots of cell area (G) and cell circularity (H) for cells within and surrounding GFP-kRas^{V12} and GFP clusters. No statistically significant differences were observed (Kruskal-Wallis test, n = mean values for 5 and 7 embryos for kRas^{V12} and GFP, respectively). (**I**) Cumulative distributions of cell shape orientation relative to cluster, 7+ cells from the cluster edge, comparing experiments (magenta) and simulations (green). Ras clusters were simulated with varying degrees of increased cortical contractility, Γ.

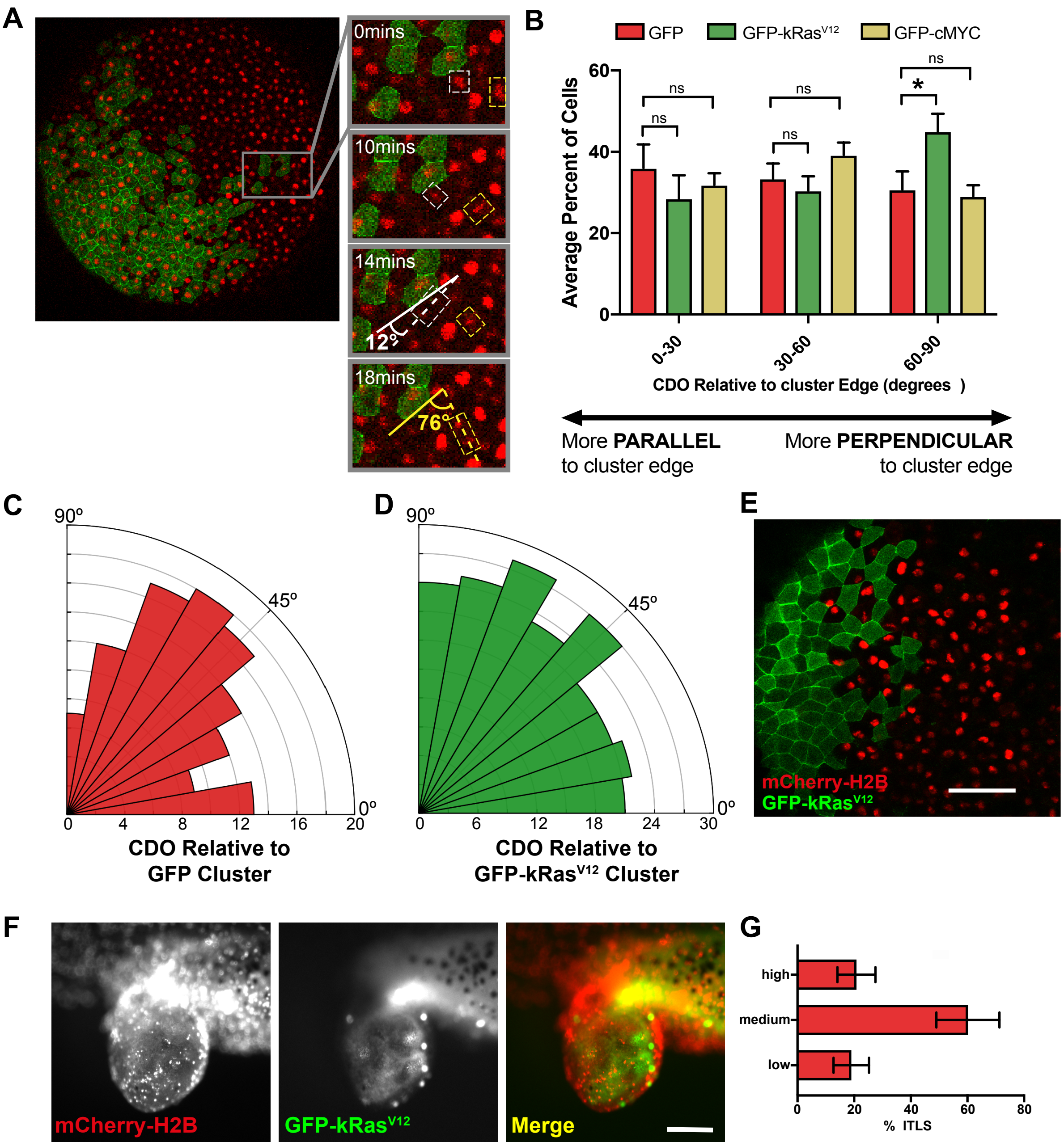
A
0mins
10mins
14mins
12°
18mins
76°
B
GFP
GFP-kRasV12
GFP-cMYC
Average Percent of Cells
60
40
20
0
ns
ns
ns
ns
ns
*
0-30
30-60
60-90
CDO Relative to cluster Edge (degrees)
More PARALLEL to cluster edge
More PERPENDICULAR to cluster edge
C
90°
45°
0°
0
4
8
12
16
20
CDO Relative to GFP Cluster
D
90°
45°
0°
0
6
12
18
24
30
CDO Relative to GFP-kRasV12 Cluster
E
mCherry-H2B
GFP-kRasV12
F
mCherry-H2B
GFP-kRasV12
Merge
G
high
medium
low
0
20
40
60
80
% ITLS

Figure S3: Further Characterisation of Wild-type Cell Behaviour in *Xenopus laevis* Embryos with Oncogene-Expressing Cell Clusters. Related to Figure 3.

(**A**) Stills from a confocal microcopy time-lapse show the quantification of cell division orientation within the epithelial plane, relative to a GFP-expressing cluster. An angle of 90° indicates a division perpendicular to the border of the cluster, whereas an angle of 0° indicates a division parallel to it. (**B**) Bar chart shows the average percentage of cell divisions in 30° bins that occurred in wild-type cells up to 3 cells from GFP, GFP-kRas^{V12} or GFP-cMYC clusters. Kruskal-Wallis test *p<0.05; n=8 GFP, 9 GFP-kRas^{V12} and 9 GFP-cMYC embryos. Error bars show SEM. (**C-D**) Rose histograms show cell division orientation, relative to the cluster edge, of wild-type cells 7+ cells from (C) control-GFP or (D) GFP-kRas^{V12} clusters, in 10° bins. Kruskal-Wallis test: p>0.9999: shows no significant difference between distributions; Chi- squared tests show no significant difference from uniform distribution for (C) or (D); n=120 divisions from 8 GFP-control embryos and 212 divisions from 11 GFP-kRas^{V12} embryos. (**E**) Confocal microscopy image of a stage 10 *Xenopus* embryo injected with GFP-kRas^{V12} (green) mRNA in a single cell at the 32-cell stage; mCherry-H2B (red) mRNA was then injected into neighbouring cells at the 32-cell stage. Scale bar is 100 µm. (**F**) Images showing a representative ITLS, in a stage 38 embryo, that had a GFP-kRas^{V12} cluster and wild-type cells labelled with cherry-H2B at stage 10. Wild-type cells (red) can be seen contributing to the ITLS. Scale bar represents 500 µm. (**G**) Categorisation of ITLS from GFP-kRas^{V12} embryos according to quantity of wild-type cells present in ITLS (ITLS in F, categorised as "high"). Error bars show SEM, n=6 independent experiments, 39 embryos.

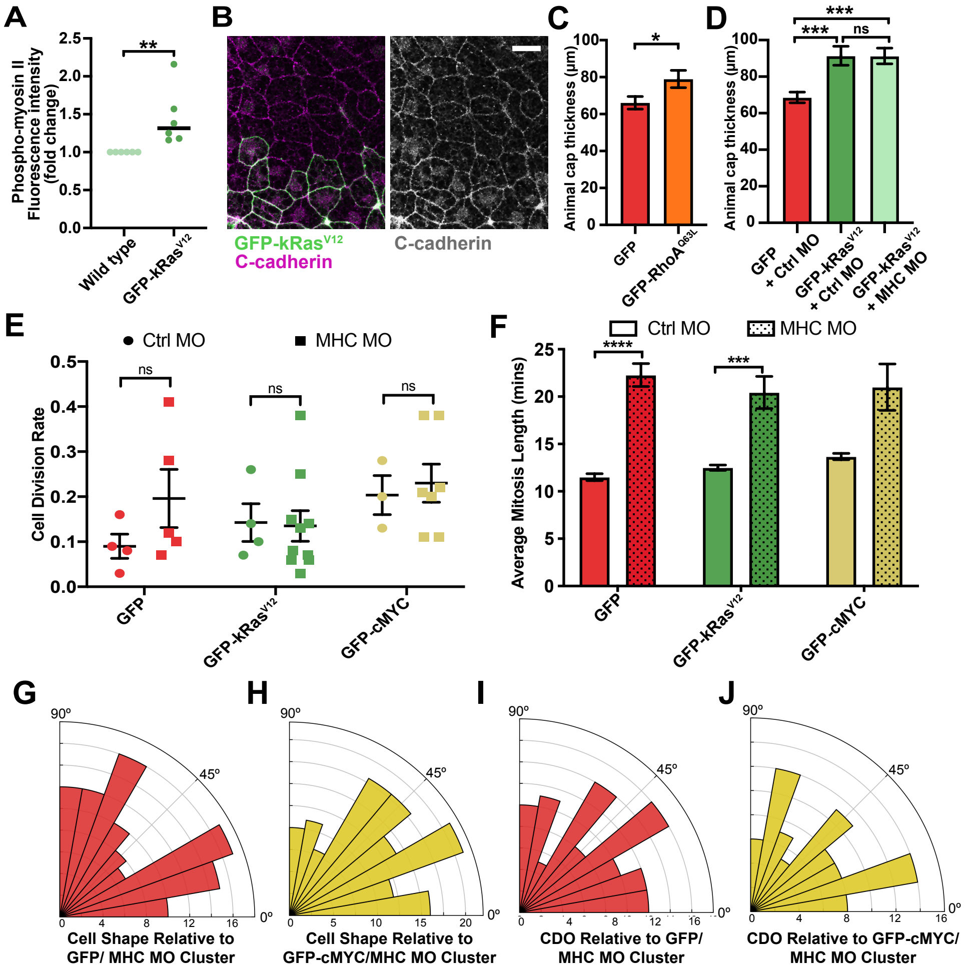
A
B
C
D
E
F
G
H
I
J
Phospho-myosin II Fluorescence intensity (fold change)
Wild type
GFP-kRas^{V12}
GFP-kRas^{V12}
C-cadherin
C-cadherin
Animal cap thickness (μm)
GFP
GFP-RhoA^{Q63L}
GFP + Ctrl MO
GFP-kRas^{V12} + Ctrl MO
GFP-kRas^{V12} + MHC MO
Ctrl MO
MHC MO
Cell Division Rate
GFP
GFP-kRas^{V12}
GFP-cMYC
Average Mitosis Length (mins)
Cell Shape Relative to GFP/ MHC MO Cluster
Cell Shape Relative to GFP-cMYC/MHC MO Cluster
CDO Relative to GFP/ MHC MO Cluster
CDO Relative to GFP-cMYC/ MHC MO Cluster

Figure S4: Further Characterisation of the Depletion of Myosin II in Oncogene-Expressing Cell Clusters. Related to Figure 4.

(**A**) Quantification of fluorescence intensity for phospho-myosin II staining in stage 10 embryos with a GFP-kRas^{V12} cluster. Fluorescence intensity in kRas^{V12} cells is shown as a fold-change compared to wild-type cells in the same embryo (*p<0.01, Mann Whitney, n=6 embryos. (**B**) Confocal slice of C-cadherin staining (magenta) in GFP-kRas^{V12} clusters (green) and surrounding wild-type tissue. Scale bar represents 20μm. (**C**) Quantification of mean animal cap thickness at GFP and GFP-RhoA^{Q63L} clusters in stage 10 embryos (p<0.05, Unpaired t-test, n = 24 and 30 embryos respectively). (**D**) Quantification of mean animal cap thickness at GFP/Ctrl MO, GFP-kRas^{V12}/Ctrl MO and GFP-kRas^{V12}/MHC MO clusters in stage 10 embryos (***p<0.001, Kruskal-Wallis, n = 24, 32 and 19 embryos respectively). (**E**) Dot plot shows the average percentage of cells per minute that divided in clusters that were co-injected with GFP, GFP-kRas^{V12} or GFP-cMYC mRNA and either control morpholino (Ctrl MO) or myosin heavy chain IIB morpholino (MHC MO). GFP: Kruskal-Wallis test: n=4 GFP/Ctrl MO embryos, 5 GFP/MHC MO, 5 kRas^{V12}/Ctrl MO and 7 kRas^{V12}/MHC MO, 3 GFP-cMYC/Ctrl MO and 4 GFP-cMYC/Ctrl MO embryos. Error bars are SEM. (**F**) Bar chart shows the average number of minutes between nuclear envelope breakdown and the separation of daughter nuclei in anaphase. Kruskal-Wallis test: ****p<0.0001, ***p=0.0007 n=12 GFP/Ctrl MO cells, 7 GFP/MHC MO, 12 kRas^{V12}/Ctrl MO, 9 kRas^{V12}/MHC MO, 12 GFP-cMYC/Ctrl MO and 4 GFP-cMYC/Ctrl MO. Error bars are SEM. (**G-H**) Rose histograms show the orientation of wild-type cell long-axes up to 3 cells or from myosin II deficient (F) GFP clusters or (G) GFP-cMYC clusters, relative to the cluster, in 10° bins. Kruskal-Wallis test performed against GFP/Ctrl MO shown in Figure 4G: GFP/Ctrl MO vs GFP/MHC MO p>0.9999, GFP/Ctrl MO vs GFP-cMYC/MHC MO p=0.0803, n=325 cells from 6 GFP/Ctrl MO embryos, 107 cells from 7 GFP/MHC MO embryos and 128 cells from 8 GFP-cMYC/MHC MO embryos. (**I-J**) Rose histograms show cell division orientation of wild-type cells up to 6 cells from myosin II deficient (H) GFP clusters or (I) GFP-cMYC clusters, relative to the cluster in 10° bins. Kruskal-Wallis test performed against GFP/Ctrl MO shown in Figure 4H: GFP/Ctrl MO vs GFP/MHC MO p>0.9999, GFP/Ctrl MO vs GFP-cMYC/MHC MO p>0.9999, n=58 divisions from 6 GFP/Ctrl MO embryos, n=99 divisions from 7 GFP/MHC MO embryos and 80 divisions from 8 GFP-cMYC MHC MO embryos.

mRNA Construct	Stage Injected	Total mRNA injected into each cell
Cherry-Histone-H2B	2-cell (both cells)	0.42 ng
Cherry-Histone-H2B	32-cell (multiple cells)	0.21 ng
BFP-CAAX	2-cell (both cells)	0.42 ng
GFP	2-cell (both cells)	0.42 ng
GFP	32-cell (one cell)	0.21 ng
GFP-kRas^{V12}	2-cell (both cells)	0.42 ng
GFP-kRas^{V12}	32-cell (one cell)	0.263 ng
GFP-cMYC	2-cell (both cells)	0.42 ng
GFP-cMYC	32-cell (one cell)	0.21 ng
GFP-RhoA^{Q63L}	32-cell (one cell)	0.105 ng

TABLE S1: List of mRNA concentrations injected into *Xenopus* embryos. Related to STAR Methods.
